# The Effect of Powder Reuse on Electron Beam Melting for Biomedical Implants

**DOI:** 10.3390/ma17194701

**Published:** 2024-09-25

**Authors:** Akshay Mundayadan Chandroth, Paula Milena Giraldo-Osorno, Lars Nyborg, Anders Palmquist, Yu Cao

**Affiliations:** 1 Department of Industrial and Materials Science, Chalmers University of Technology, 41296 Gothenburg, Sweden; akshay.chandroth@kuleuven.be (A.M.C.); lars.nyborg@chalmers.se (L.N.); 2 Department of Materials Engineering, KU Leuven, Kasteelpark Arenberg 44 bus 2450, 3001 Leuven, Belgium; 3 Department of Biomaterials, University of Gothenburg, 41346 Gothenburg, Sweden; paula.giraldo@biomaterials.gu.se (P.M.G.-O.); anders.palmquist@biomaterials.gu.se (A.P.)

**Keywords:** additive manufacturing, Ti-6Al-4V, microstructure, bone implants, powder recycling

## Abstract

The ability of additive manufacturing to generate intricate structures has led to its popularity and widespread use in a variety of applications, ranging from the production of biomedical implants to aircraft components. Additive manufacturing techniques can overcome the limitations of the traditional manufacturing methods to create complex near-net-form structures. A vast array of clinical applications effectively employ Ti-6Al-4V as a biomaterial. The evolution of additive manufacturing has accelerated the development of patient-specific implants. The surface characteristics play a critical role in tissue healing and adaptation to implants. The present research set out to examine the effects of powder recycling with respect to the powder itself and the surface properties resulting from the electron beam melting (EBM) of the implant material. The printed implants, as well as the powder samples, underwent morphological, surface chemistry, and microstructure analyses. The in vitro cytotoxicity was evaluated with THP-1 macrophages. The overall microstructure of the implant samples showed little variation in terms of powder recycling based on the results. Higher oxygen levels were found in the solid and lattice sections of those implants manufactured with batches of recycled powder, along with marginally better cell viability. This emphasizes how crucial powder quality is to the process of additive manufacturing.

## 1. Introduction

Additive manufacturing (AM), especially powder-based technology, makes the fabrication of complexly shaped metal implants possible. It is quickly becoming a standard production method for biomedical applications. To continually improve the fabrication process and produce the greatest components, several parameters like implant chemistry and morphology must be considered [1,2].

One of the most popular AM methods for producing metal implants is electron beam melting (EBM), which is being clinically used in a wide range of anatomical locations to replace anatomical parts and restore musculoskeletal functions [3]. Due to its effectiveness, it is capable of printing complex thin structures and could rapidly fabricate complex components. EBM-related research has been receiving a great deal of attention in recent times. In this manufacturing process, complicated shapes are produced based on the stereolithographic (STL) input of the design via the layer-by-layer fusion of metal powders using an electron beam. Due to the use of high-intensity electron beams, the lead times of this AM technique can be greatly shortened when compared to the laser-based approach.

Ti-6Al-4V is the most widely used titanium alloy for biomedical implants. The alloy’s superior corrosion resistance and strength to weight ratio and a similar elastic modulus to human bone make it an appropriate choice for developing implants [4]. The preclinical evaluation of the Ti-6Al-4V implants produced by EBM shows high potential, where the as-produced surface enables bone growth and maturation [3]. It is widely known that surface topography and surface oxide are important for bone healing around implants, and continuous work in altering the as-EBM surface structure is currently ongoing to further boost bone healing [5,6]. One of the technologies to alter the implant surface could be micro-alloying, where a tailored surface composition could be obtained that improves the bone anchorage [5]. 

In previous investigations, the powder reuse times were evidently shown to influence the tensile properties of the EBM-printed Ti-6Al-4V in a beneficial way due to the increase in oxygen content [7]. However, for biomedical applications, there is a great lack of knowledge on the surface characteristics, net structure, and design of the implants, as well as the effect of powder reuse in most preclinical and clinical studies. The current study aimed to characterize the effect of powder reuse both in terms of changes in the powder and the built parts, with an emphasis on the microstructure, chemistry, porous lattice structure, and surface characteristics, as well as cell cytotoxicity.

## 2. Materials and Methods

### 2.1. Materials and Building Conditions

Commercial Ti-6Al-4V powder with an average particle size of approximately 50 μm produced by plasma atomization was used in this investigation. A standard build protocol was used. Acetabular cups and rod-shaped samples were built by a GE Additive Q10 plus system (Arcam AB, Gothenburg, Sweden) using both virgin and recycled powders. Prior to each run, non-used powders were collected, sieved, and then reused for the subsequent run. Powder samples in the state of virgin and 5-time recycling, together with corresponding built samples, were characterized.

### 2.2. Sample Preparation 

For surface characterization, powders were pressed slightly on a thin pure copper sheet, while the scaffold samples were cut into small pieces at different distances from the build plate. The samples for cell cultures were cut from the printed rod into pieces suitable for a 12-well plate prior to sterilization in ethanol. For microstructural analyses, powders and pieces from the solid and lattice regions of the scaffold were mounted with Polyfast and then ground with 1200 SiC grit paper, followed by fine polishing using an OP-S solution (90% OP-S and 10% H_2_O_2_). After being cleaned in an ultrasonic bath for 20 to 25 min using isopropanol and ethanol, the polished samples were etched with Kroll’s reagent (1–2% HF + 3% HNO_3_ + H_2_O).

### 2.3. Powder Characterization

The surface morphology and microstructural aspects of powders were examined by scanning electron microscopy (SEM) using a Ziess Leo Gemini FEG-SEM (Zeiss, Oberkochen, Germany) operating at 10 kV with an aperture size of 30 μm. The size distribution of the powders (PSD) was statistically analyzed by ImageJ software V 1.53 from ~1000 different powder particles on the SEM images. An Oxford X-mas EDS detector (Oxford Instruments, Oxford, UK) connected to the SEM was used to analyze the chemical composition of ten different powder particles at an acceleration voltage of 10 kV at a fixed working distance of 8.5 mm with an aperture size of 60 μm.

### 2.4. Scaffold Characterization 

Internal porosity and defects were evaluated using a Zeiss Axioscope 7 light optical microscope (Zeiss, Oberkochen, Germany). The integration function provided by Zeiss Zen Core 2.7 software was used to stitch the images to obtain a large field of view. The relative density of the printed samples was averaged from 12 stitched images with a magnification of 500×. The microstructure was evaluated by SEM after etching.

Surface chemistry was analyzed by X-ray photoelectron spectroscopy (XPS) on the solid region and the porous lattice region of the sample using a PHI 5000 VersaProbe III (Physical Electronics, Chanhassen, MN, USA) equipped with a monochromated Al Kα X-ray source with an energy of 1486.6 eV using X-ray beam size of 100 µm and take-off angle of 45°. A survey scan was conducted in the binding energy range of 0–1100 eV. To determine element distribution in depth, alternating XPS measurements were conducted covering Ti 2p, Al 2p, V 2p_3/2_, C1s, O1s, and N1s, and argon ions sputtering with a raster size of 2 × 2 mm and an ion beam voltage of 2 kV. The etch rate calibrated by Ta_2_O_5_/Ta with known oxide thickness was 52 Å/min. The oxide thickness was defined as the depth when the intensity of oxygen decreased by half.

### 2.5. In Vitro Characterization

#### 2.5.1. THP-1 Cell Expansion and Differentiation

THP-1 human monocytic cell line THP-1 (ATCC TIB-202, Manassas, VA, USA) was grown in Roswell Park Memorial Institute 1640 medium (RPMI) supplemented with 10% fetal bovine serum, 0.5% β-mercaptoethanol (Sigma Aldrich, Munich, Germany) and 1% penicillin/streptomycin solution (Gibco Life Technologies, Carlsbad, CA, USA). The cells were grown in 75 cm^2^ culture flasks at 37 °C in a humidified incubator with 5% CO_2_. Cells in passage 4 were used, and the RPMI medium was refreshed every two days. To induce macrophage differentiation, THP-1 monocytes were stimulated with 10 ng/mL phorbol-12-myristate-13-acetate (PMA, Sigma Aldrich, Munich, Germany) for 48 h, followed by 24 h of resting time in fresh RPMI without PMA.

#### 2.5.2. THP-1 Macrophage Seeding

Macrophages were detached using trypsin (Gibco Life Technologies, WA, USA), and 250,000 cells were drop-seeded onto the surface of each material in Nunc 12-well plates (Thermo Fisher Scientific, Roskilde, Denmark). After 1 h of direct cell contact, each well was completed with 3 mL RPMI media and left for 24 h culture.

#### 2.5.3. Cell Adhesion and Viability 

The number of viable adhered cells on the surface was quantified using a NucleoCounter^®^ NC-200TM system (ChemoMetec A/S, Lillerød, Denmark) following the manufacturer’s instructions. In brief, to quantify the total number of cells adhered, 250 µL of the NucleoCounter^®^ lysis buffer and 250 µL of the stabilization buffer were added directly to the disks and subsequently loaded into a Nucleocassette™ (ChemoMetec A/S, Lillerød, Denmark).

To further assess cytotoxicity, the enzymatic activity of cytosolic lactate dehydrogenase (LDH) released into the cell culture supernatants was measured using the CyQUANT-™ LDH Cytotoxicity Assay (Thermo Fisher Scientific, Roskilde, Denmark) following the manufacturer’s protocol. In brief, after 24 h of direct contact with the materials, 50 µL of supernatant from each sample was plated in Nunc 96-well plates (Thermo Fisher Scientific, Roskilde, Denmark), and 50 µL of substrate from the LDH assay kit was added to each well. Absorbance was read at 490 nm every 1 min for 30 min using a FLUOstar Omega Microplate reader (BMG LABTECH, Ortenberg, Germany).

#### 2.5.4. Cell Attachment and Morphology

To evaluate the impact of recycling on cell morphology and adherence, macrophages that had adhered to both virgin and recycled materials for 24 h, as previously described, were fixed for 15 min using a 4% formaldehyde solution (HistoLab AB, Askim, Sweden), followed by two washes with PBS. Subsequently, the cells adhered to the materials were stained with phalloidin (ActinRed™ 555 ReadyProbes™ Reagent, Rhodamine phalloidin, Invitrogen, Waltham, MA, USA), which selectively binds to F-actin and serves as a marker for total adhered cells. Nuclei were stained with DAPI (Ibidi, Fitchburg, WI, USA) in preparation for imaging using a Nikon C2+ confocal laser-scanning microscope (CLSM; Nikon, Tokyo, Japan).

## 3. Results

### 3.1. Powder Samples

**Figure 1:**
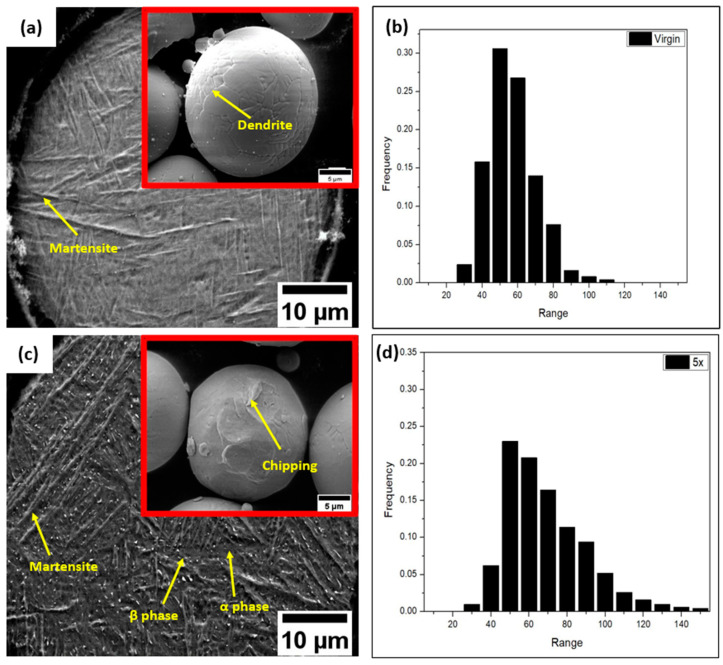
Images portraying the surface morphology and microstructure of the virgin powder (**a**) and (**b**) 5 times-recycled powder; (**c**) PSD of virgin powder (**d**) and 5-times-recycled powder.

The virgin powder was presented with a smooth surface and high sphericity with only minor small satellite particles (insert in Figure 1a). Clear dendritic features can be observed on the powder surface, which are evident from the branched pattern. The recycled powder, on the other hand, showed a rougher and sometimes deformed and chipped surface structure without dendrite (insert in Figure 1c).

A rather homogenous microstructure dominated by needle-like acicular martensite ranging in size from 2 to 40 μm was revealed for the virgin powder, while a mixed microstructure was found after recycling (Figure 1a and Figure 1c). In addition to the needle-like acicular martensite phase, it encompassed bright white particulate that was identified as β phases by EDS analysis. Furthermore, bulge-like structures, expected to be an α phase, were also observed [8,9]. The recycling broadened the powder size distribution (Figure 1b,d) toward a larger size. However, the particles were still mainly in the range of 40–100 μm.

To examine the composition of the powders in the bulk and at the surface, EDS analysis was performed on both cross-sections, which provided the information of the bulk, and on the surface of the particles, which was more surface-related. As shown in Table 1, oxygen could be detected by EDX. Notice that EDS faces challenges when it comes to quantifying light elements such as oxygen. It has been well known that oxygen has a high solubility in Ti. This may cause oxygen to dissolve in the extreme surface region. Together with possible surface oxidation during sample preparation, a higher oxygen content is observed compared to the nominal composition. However, the values in the table still provide indicative information. An increase in the oxygen content was observed for the recycled powder both at the surface and in the bulk. Another observation was that the surface had higher Al but lower V content for both the virgin and recycled powders. 

### 3.2. Scaffold Samples

#### 3.2.1. Microstructure and Elemental Composition

**Figure 2:**
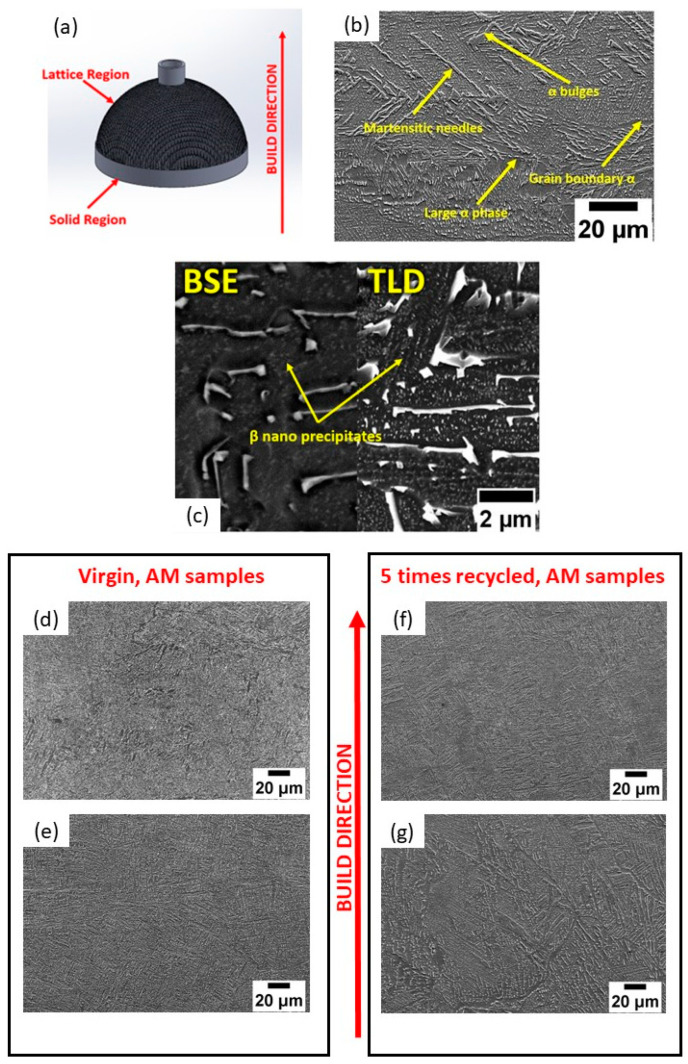
(**a**) Model of AM-built sample portraying the solid and lattice regions of the sample; (**b**) typical microstructure (SE image) of virgin AM sample showing different features; (**c**) TLD (Through the Lens Detector) (**right**) and BSE (Backscattered Electron) (**left**) images of 5-times-recycled AM samples; (**d**,**e**) microstructure (SE images) of AM samples using virgin powders at different locations; (**f**,**g**) microstructure (SE images) of AM samples using 5-times-recycled powder at different locations. The building direction is marked in the figure, and (**e**,**g**) are near the build plate.

The built scaffold sample and corresponding microstructure are shown in Figure 2. Although the virgin and recycled powder particles used as feedstock differed from one another to a certain extent, there was no significant influence on the evolved microstructures. The samples fabricated with these two powders had similar microstructures and demonstrated similar trends in microstructural variation with respect to the building height.

A typical microstructure observed in the EBM Ti-6Al-4V is provided in Figure 2b. Aside from the large needle-shaped irregularly oriented martensite, as marked in the figure, colony and basketweave microstructures consisting of alpha (α) and beta (β) phases were observed. The bright features were β phases spread in a dark α phase matrix [10]. These two phases tended to obey Burger’s orientation relationship [8,11]. Another prevalent phase found throughout the microstructure was a huge black phase recognized as α from the compositional analysis. It was marked as the α bulge in Figure 2b [12]. In addition, the prior β grain boundary [8,13] was also observed. In all the samples, a distinct nanosized feature was observed in the α phase matrix (Figure 2c). It was reported to be Ti_3_Al precipitates from the previous studies [12,14]. However, its bright contrast in the BSE image (Figure 2c) suggested the enrichment of elements with larger atomic numbers. This led to the possibility that these nanoparticles could be β precipitates, which are enriched by V with a larger atomic number. But, further analysis is required in the future. 

The observed microstructure is building-height-dependent. A clear difference was observed at different locations. Figure 2e,g provide the microstructure at the solid region near the build plate. Figure 2d,f provide the microstructure at the lattice region away from the build plate. Higher fractions of martensitic needles and grain refinement were found at the location far from the build plate (Figure 2d,f). This observed heterogeneous microstructure is caused by the complicated thermal behavior of the manufacturing process. However, the fraction of martensite is small in general.

The EDS examination of the various phases within the microstructure of the virgin and five-times-recycled implants (Table 2) discloses the presence of elements like Ti, Al, and V, which is comparable to the nominal composition of the alloy [15]. From the analysis, it was found that the α phase was aluminum-enriched and the β phase was vanadium-enriched. It should be noted that there were no considerable differences in the chemical composition of the phases in the solid and lattice microstructures with powder recycling.

#### 3.2.2. Porosity

**Figure 3:**
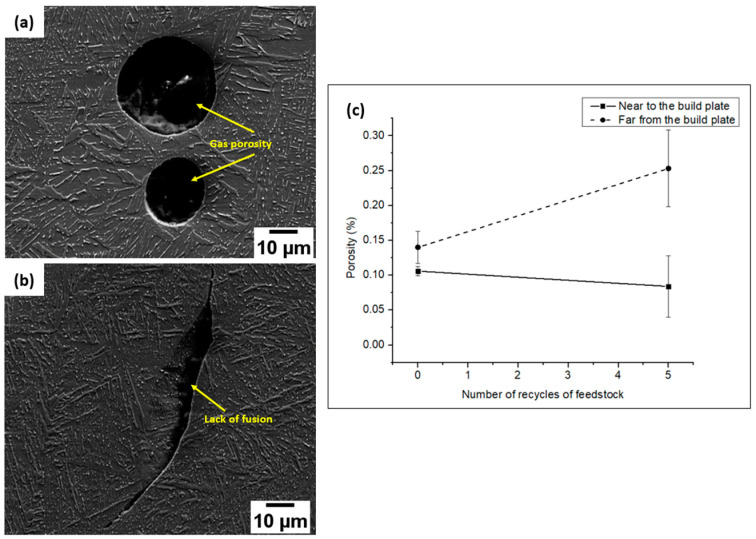
Images portraying (**a**) gas porosity (SE image), (**b**) lack of fusion defects (SE image), and (**c**) graphical representation of porosity percentage variation with respect to variation in build height and powder recycle number.

Typical EBM defects, such as a lack of fusion and gas porosities, were revealed in the AM samples using virgin and 5-times-recycled (Figure 3a,b) powders. The sizes of the porosities ranged between 2 and 80 μm. Figure 3c displays the porosity percentages at different locations. For both the AM samples manufactured using the virgin and 5-times-recycled powders, there was a clear increase in porosity with increasing build height due to the intricate thermal gradient. 

At the region further from the build plate, the porosity value of the AM sample produced using recycled powder demonstrated an obvious increment compared to the one using the virgin powder. However, the difference was small close to the build plate. It should be mentioned that small pores were skipped in the porosity evaluation due to the resolution limitation (under 500 magnification).

#### 3.2.3. Surface Chemistry of the Solid and Lattice Regions of the Implant Samples

**Figure 4:**
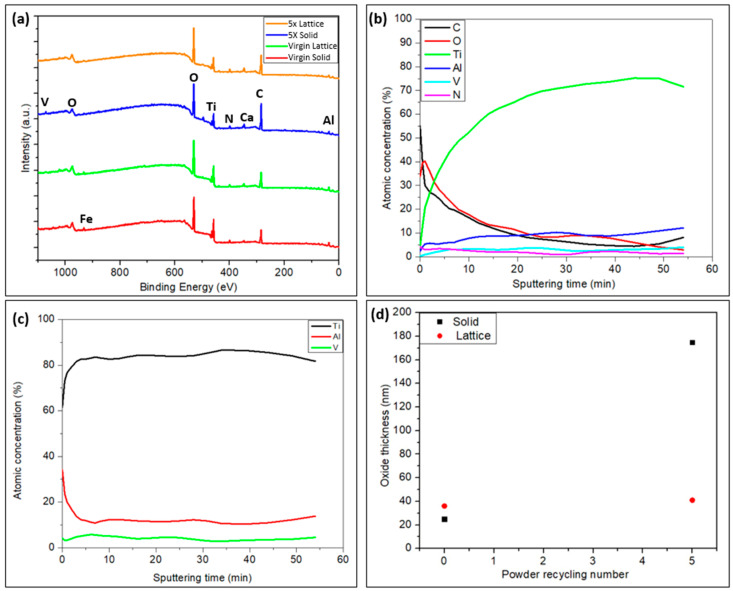
Graphs portraying the (**a**) survey spectra of all the solid and lattice samples; (**b**) depth profiles of 5-times-recycled AM lattice samples; (**c**) cation depth profiles of 5-times-recycled AM lattice samples; (**d**) oxide thickness of solid and lattice regions in virgin and 5-times-recycled Am samples. The etch rate calibrated by Ta_2_O_5_/Ta with known oxide thickness was 52 Å/min.

The presence of elements such as Ti, V, Al, O, C, Ca, and N was disclosed by the XPS survey spectrum at the surface of both the solid and lattice regions (see Figure 2a for the analysis location) of the implants (Figure 4a). In addition to the peaks from these elements, an Fe2p core level with a low intensity was also detected in both the lattice and solid regions of the samples manufactured using the virgin powder. However, it was less clear for the sample manufactured using the recycled powder. This might be due to the increased peak intensity of C, which is typical surface contamination. The presence of C attenuates the photoelectrons from Fe2p, thus suppressing the detection of Fe in the recycled powders. 

As shown in a typical depth profile in Figure 4b, the concentration of Ti dramatically increases towards the interior regions of the solid and lattice regions of the implant samples irrespective of the feedstock. A similar pattern was observed in the concentration levels of Al and V; even so, the concentration of V increased in a negligible manner. This was consistent with the high concentrations of O and C at the surface. Carbon was adventitious. The variation in the O concentration with respect to the depth or etch time is noteworthy. It was higher at the surface and declined afterwards, indicating the existence of oxide at the surface in all the cases. For comparison purposes, the oxide thickness was defined as the depth when the oxygen intensity (O1s) was reduced by half. The results are provided in Figure 4d. For the lattice region, using the recycled powder slightly increased the oxide thickness on the manufactured part from 38 to 40.5 nm compared to the virgin powder. Surprisingly, for the solid region, there was a clear rise in the oxide thickness in the sample fabricated using the 5-times-recycled powder, as revealed in Figure 4d. This might reflect uneven oxide thickness, i.e., varied oxide layer thickness at different locations of the samples. Nevertheless, when the recycled powders were used, the experimental observation above implied a general increased oxide thickness, at least locally.

To clarify the possible surface segregation of the elements of interest, the cation depth profile was calculated, as shown in Figure 4c. Here, only Ti, Al, and V were included in the quantification. For both the solid and lattice regions from the samples prepared using the virgin and five-times-recycled powders, the enrichment of Al at the surface was observed from the cation depth profile owing to the strong affinity of Al with oxygen. This was in accordance with the EDX results in Table 1, where the Al concentration was discovered to be decreasing in the inner region. 

#### 3.2.4. THP-1 Macrophage Viability and Adhesion

**Figure 5:**
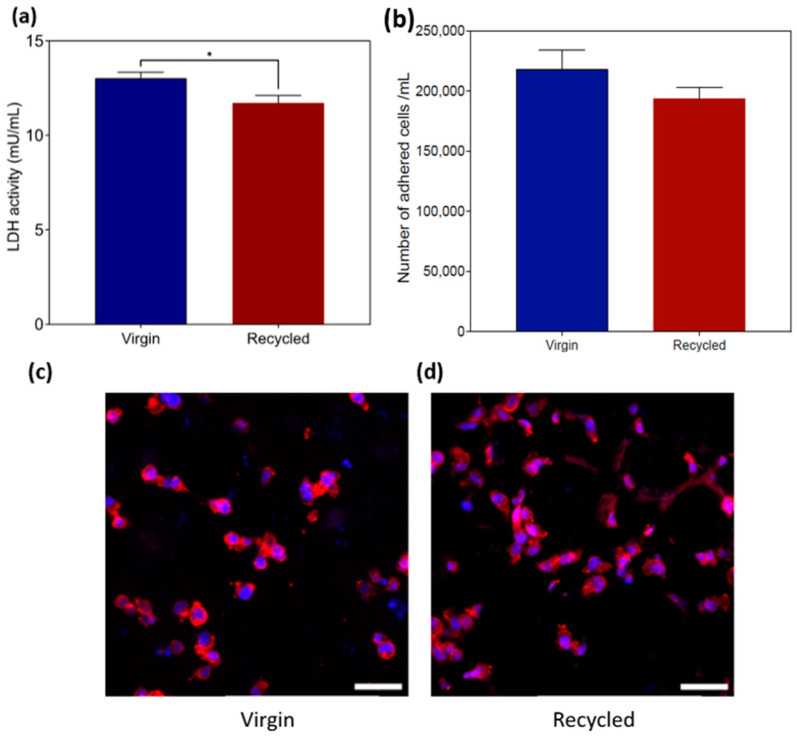
(**a**) LDH activity after 24 h of direct contact; (**b**) number of viable THP-1 macrophages adhered to the surfaces; cell adhesion, cell nuclei stained with DAPI (blue), and F-actin stained with ActinRed (red) imaged by confocal laser scanning microscopy 24 h after seeding into (**c**) virgin; (**d**) recycled. Data represent the means from three independent experiments (n = 3; ±SD) with duplicate samples. * *p* ≤ 0.01 using *t*-test. Scale bar = 50 µm.

The release of intracellular LDH into the culture medium was quantified to assess the cell death attributed to membrane damage resulting from direct contact with the materials. The LDH release concentration for both virgin and recycled was lower than 15 mU/mL per 1 × 10^5^ cells, indicating good biocompatibility. Neither material showed signs of membrane damage (Figure 5a), although a significantly lower value for the recycled powder was observed. Moreover, there was no statistically significant difference in the number of viable cells adhering to both virgin and recycled (Figure 5b). 

To evaluate whether the materials had any impact on the morphology of the adhered macrophages, the cells were stained to visualize their nuclei and F-actin filaments. No visible differences were observed between the groups after a 24 h interaction, suggesting that the materials do not influence cell adhesion. Across all the groups, the cells were observed to spread homogenously along the material surfaces. The most prevalent morphology consisted of small rounded cells (Figure 5c,d). 

## 4. Discussion

From the surface morphological studies of the feedstock, it is evident that the powders underwent severe surface deterioration as a result of the thermal degradation and repeated recycling process [16], which can cause rough patches and chipping on the powder surface. The lack of a satellite can be attributed to the blasting and sieving in the recycling process, which breaks down the nanoscale agglomerates [9,16]. The powder shapes have also been observed to vary. The sphericity of powders is critical in determining the powder layer thickness and uniformity, thus possibly affecting the build quality and density of the implant samples. This could be observed from the varied porosity percentages in the recycled and virgin AM samples (Figure 1c,d). Particle size distribution (PSD) is a crucial component that can dramatically change the porosity percentage and dimensions of the evolved pores. It affects the powder layer thickness, likely resulting in imperfect melting and the establishment of process-induced gas porosities, which were observed in the recycled powder batches [9,17].

The variation in the microstructure is an interesting observation to be noted in the virgin and recycled powder samples. Due to the sudden rapid quenching of the atomized drops in the molten state by the flow of gas during the Gas Atomization process, a higher martensitic fraction and inner porosities are visible in the virgin powder [9]. The presence of various α and β phases in the recycled powder samples is attributed to the complicated thermal history of the powder samples. The recycling process can also tailor the surface elemental composition of the powder. Although it is complicated to quantify light elements like oxygen by EDX [18], the observed increase in the O concentration could be attributed to an effect of recycling. The higher concentration of Al observed on the powder surface is an interesting finding in this study. Aluminum has a high oxygen affinity and tends to migrate from the interior to the surface of the powder to form Al_2_O_3_ [19]. This could explain the difference in the aluminum content at the surface versus the lower concentration at the interior, as observed with EDS in Table 1. The existence of the evaporation of the aluminum from the powder samples during the repetitive melting and recycling process is confirmed by previous studies [19,20]. This occurs in a fairly shallow region, and further XPS analysis on the powder surface is essential for validation.

The microstructures of the AM samples fabricated using both powder batches show trivial variations with each other. Features like martensite, α, and β phases are exhibited. Burger’s orientation relationship is also noticed in the martensitic needles found in the microstructure [9,21]. The microstructure of the starting powder has minimal effects on the final microstructure of the printed parts. This can be viewed as a positive effect, implying minimal variation in the microstructure-dependent mechanical properties of the implants with respect to powder recycling. Thus, from a sustainable point of view, powder recycling can be beneficial. However, variations can be observed in the microstructures of the parts depending on the building height due to the varied cooling rates and scanning strategies at the region far from the build plate. Although more martensite is observed at the top of the builds, in general, the fraction of martensite is small. The existence of martensite may affect the fatigue performance negatively. Nevertheless, post-processing such as HIP could be employed to remove the localized fraction of martensite. The porosity percentage of the samples is also observed to exhibit a variation based on the difference in build height. These defects are typically the consequence of process anomalies such as elevated beam temperature and trapped gases during the laser-based fusion of powders, and they can usually be minimized by tailoring the process parameters [22,23]. 

From the surface chemical analysis of the implant samples, C and Ca were observed, and they are supposed to be contaminants existing on the implant surfaces [20,24] as the intensity of these peaks declined instantaneously by Ar+ ion sputtering. Repeated recycling causes an increase in the O concentration in the powder, as observed with EDS. In general, the oxide thickness is higher when using recycled powder, at least locally (Figure 4d). This is consistent with the findings of other researchers [25,26]. It has been reported that an increase in the O concentration in the feedstock material results in a similar increment of oxide thickness at a nanometric level in the implant samples. As a consequence, the bone response to implants is enhanced [27,28]. The cell viability, as judged by LDH, showed a significant improvement with the increased oxide thickness without altering the cell attachment and morphology. In the present study, we evaluated the early interaction by inflammatory cells, which are crucial for the communication of subsequent healing events. Further studies using more sophisticated co-culture models, including the communication between the inflammatory cells and regenerative cells, are needed to understand the exact mechanisms of the role of surface oxide. 

In previous studies, the mechanical properties of the EBM-printed Ti-6Al-4V were investigated [29,30]. It was found that the implants printed with reused powder have a lower fatigue life (~10^4^–10^6^ cycles) than the required standards for biomedical implants (~10^5^–10^7^ cycles) due to the increase in the surface oxide layer and higher fraction of defects. However, simple post-processing techniques like Hot Isostatic Processing (HIP) could solve this issue to a reliable extent. Considering the sustainability and economic aspects, the other benefits of using reused powder for the EBM fabrication of implants far outweigh this reduction in mechanical properties. Thus, this study shows that there is significant potential for using reused powders to manufacture bioimplants, reducing the wastage of valuable resources. 

## 5. Conclusions

The purpose of this study was to clarify the effect of powder recycling on the microstructural and chemical properties of powder samples and AM-manufactured parts. The cell viability and adhesion were also evaluated. From the detailed assessments of the feedstock and printed implant samples, the following conclusions were formulated.

1. The powder particles were distorted, and the surface quality was found to be degenerated after recycling.
2. The martensite percentage in the virgin powder was relatively high. After recycling five times, the powder particles’ microstructure changed visibly. Various microstructural features, like α bulges, a large α phase, and β phases, were discovered in the five-times-recycled powders. Also, the EDS results indicate that the surface area had a greater concentration of Al than the interior region of the powder. The O concentration seemed to be higher with powder recycling.
3. Apart from the conventional Ti-6Al-4V phases, unconventional α bulges, large patches, and β nanoparticles were revealed in the microstructure. In addition, the bottom and top sections along the building direction of the AM specimens differed to a certain extent. However, there is a trivial variation with respect to powder recycling.
4. The porosity evaluations uncovered the presence of classic EBM flaws, such as a lack of fusion and gas porosities. All the implant samples exhibited a rise in the porosity fractions as the build height rose.
5. The implant samples created with recycled powder showed a rise in oxide thickness in both the solid and lattice regions, at least locally, attributed to the higher O concentration observed in the recycled powder due to the repeated recycling process. This seems to be beneficial for implant–tissue interaction. In addition, the enrichment in the Al at the surface points out the possibility of the formation of aluminum oxides at the surface.
6. The AM parts fabricated with virgin and recycled powders exhibited cell compatibility. A slight improvement was demonstrated by the parts manufactured with recycled powder due to the increase in surface oxide thickness.

## Figures and Tables

**Table 1:** Powder chemical composition wt-% (bulk and surface of the powder; ETH 10 kV; WD = 8.5 mm).

Powder/Region	Ti	Al	V	O
Virgin/Bulk	87.67 ± 0.52	5.97 ± 0.19	3.87 ± 0.36	2.19 ± 0.20
Virgin/Surface	86.95 ± 0.63	6.45 ± 0.20	3.63 ± 0.28	2.64 ± 0.50
5 times/Bulk	87.21 ± 0.96	5.6 ± 0.21	3.85 ± 0.63	2.62 ± 1.1
5 times/Surface	86.37± 1.51	6.72 ± 0.54	3.14 ± 0.48	3.4 ± 1.13

**Table 2:** Implant chemical composition wt-% (solid region of the AM samples; ETH 10 kV; WD = 8.5 mm).

Sample/Phase	Ti	Al	V	O
Virgin/α phase	88.88 ± 1.07	5.55 ± 0.56	3.39 ± 1.43	2.08 ± 0.31
Virgin/β phase	74.91 ± 8.10	2.89 ± 1.22	17.99 ± 8.33	1.5 ± 0.78
5 time/α phase	86.71 ± 5.39	5.89 ± 1.04	4.85 ± 5.63	1.94 ± 0.27
5 time/β phase	72.51 ± 5.50	2.74 ± 1.22	19.76 ± 6.11	1.76 ± 1.11

## Data Availability

The authors confirm that relevant data to support this research is available with in the article.

## References

[1] Millis D. (2004). Responses of Musculoskeletal Tissues to Disuse and Remobilization. Canine Rehabilitation & Physical Therapy.

[2] Tofail S.A.M., Koumoulos E.P., Bandyopadhyay A., Bose S., O’Donoghue L., Charitidis C. (2018). Additive manufacturing: Scientific and technological challenges, market uptake and opportunities. Mater. Today.

[3] Shah F.A., Snis A., Matic A., Thomsen P., Palmquist A. (2016). 3D printed Ti6Al4V implant surface promotes bone maturation and retains a higher density of less aged osteocytes at the bone-implant interface. Acta Biomater..

[4] Smoljanić T., Milović L., Sedmak S., Milovanović A., Čolić K., Radaković Z., Sedmak A. (2024). Numerical Investigation of Fatigue Behavior in Ti-6Al-4V Orthopedic Hip Implants Subjected to Different Environments. Materials.

[5] Stenlund P., Omar O., Brohede U., Norgren S., Norlindh B., Johansson A., Lausmaa J., Thomsen P., Palmquist A. (2015). Bone response to a novel Ti–Ta–Nb–Zr alloy. Acta Biomater..

[6] Grigoriev S., Peretyagin N., Apelfeld A., Smirnov A., Yanushevich O., Krikheli N., Kramar O., Kramar S., Peretyagin P. (2022). Investigation of MAO Coatings Characteristics on Titanium Products Obtained by EBM Method Using Additive Manufacturing. Materials.

[7] Tang H.P., Qian M., Liu N., Zhang X.Z., Yang G.Y., Wang J. (2015). Effect of Powder Reuse Times on Additive Manufacturing of Ti-6Al-4V by Selective Electron Beam Melting. JOM.

[8] Duda S. (2020). Microstructure Evolution of EBM Fabricated Ti-6Al-4V. Master’s Thesis.

[9] Shanbhag G., Vlasea M. (2020). The effect of reuse cycles on Ti-6Al-4V powder properties processed by electron beam powder bed fusion. Manuf. Lett..

[10] Toh W.Q., Wang P., Tan X., Nai M.L.S., Liu E., Tor S.B. (2016). Microstructure and Wear Properties of Electron Beam Melted Ti-6Al-4V Parts: A Comparison Study against As-Cast Form. Metals.

[11] Ding R., Guo Z. (2004). Microstructural evolution of a Ti–6Al–4V alloy during β-phase processing: Experimental and simulative investigations. Mater. Sci. Eng. A.

[12] Lee D.-G., Lee S., Lee C.S. (2003). Quasi-static and dynamic deformation behavior of Ti–6Al–4V alloy containing fine α2-Ti3Al precipitates. Mater. Sci. Eng. A.

[13] de Formanoir C., Martin G., Prima F., Allain S.Y., Dessolier T., Sun F., Vivès S., Hary B., Bréchet Y., Godet S. (2018). Micromechanical behavior and thermal stability of a dual-phase α+α’ titanium alloy produced by additive manufacturing. Acta Mater..

[14] Carreon H., Carreon M., Dueñas A. (2016). Assessment of precipitates of aged Ti-6Al-4V alloy by ultrasonic attenuation. Philos. Mag..

[15] Tang H.P., Zhao P., Xiang C.S., Liu N., Jia L. (2018). Ti-6Al-4V orthopedic implants made by selective electron beam melting. Titanium in Medical and Dental Applications.

[16] Ghods S., Schultz E., Wisdom C., Schur R., Pahuja R., Montelione A., Arola D., Ramulu M. (2020). Electron beam additive manufacturing of Ti6Al4V: Evolution of powder morphology and part microstructure with powder reuse. Materialia.

[17] Sun Y., Aindow M., Hebert R.J. (2017). The effect of recycling on the oxygen distribution in Ti-6Al-4V powder for additive manufacturing. Mater. High Temp..

[18] Self J., Aiken C.P., Petibon R., Dahn J.R. (2015). Survey of Gas Expansion in Li-Ion NMC Pouch Cells. J. Electrochem. Soc..

[19] Cao Y., Delin M., Kullenberg F., Nyborg L. (2020). Surface modification of Ti-6Al-4V powder during recycling in EBM process. Surf. Interface Anal..

[20] Axelsson S. (2012). Surface Characterization of Titanium Powders with X-ray Photoelectron Spectroscopy. Master’s Thesis.

[21] Tang H.P., Wang J., Song C.N., Liu N., Jia L., Elambasseril J., Qian M. (2017). Microstructure, Mechanical Properties, and Flatness of SEBM Ti-6Al-4V Sheet in As-Built and Hot Isostatically Pressed Conditions. JOM.

[22] Galarraga H., Lados D.A., Dehoff R.R., Kirka M.M., Nandwana P. (2016). Effects of the microstructure and porosity on properties of Ti-6Al-4V ELI alloy fabricated by electron beam melting (EBM). Addit. Manuf..

[23] Goel S., Ahlfors M., Bahbou F., Joshi S. (2018). Effect of Different Post-treatments on the Microstructure of EBM-Built Alloy 718. J. Mater. Eng. Perform..

[24] Watts J.F., Wolstenholme J. (2003). An Introduction to Surface Analysis by XPS and AES.

[25] Yadroitsava I., Plessis A.D., Yadroitsev I. (2019). Bone regeneration on implants of titanium alloys produced by laser powder bed fusion: A review. Titanium for Consumer Applications.

[26] Ataee A., Li Y., Fraser D., Song G., Wen C. (2018). Anisotropic Ti-6Al-4V gyroid scaffolds manufactured by electron beam melting (EBM) for bone implant applications. Mater. Des..

[27] Larsson C., Thomsen P., Lausmaa J., Rodahl M., Kasemo B., Ericson L. (1994). Bone response to surface modified titanium implants: Studies on electropolished implants with different oxide thicknesses and morphology. Biomaterials.

[28] Larsson C., Thomsen P., Aronsson B.-O., Rodahl M., Lausmaa J., Kasemo B., Ericson L. (1996). Bone response to surface-modified titanium implants: Studies on the early tissue response to machined and electropolished implants with different oxide thicknesses. Biomaterials.

[29] Popov V.V., Katz-Demyanetz A., Garkun A., Bamberger M. (2018). The effect of powder recycling on the mechanical properties and microstructure of electron beam melted Ti-6Al-4 V specimens. Addit. Manuf..

[30] He Y., Burkhalter D., Durocher D., Gilbert J.M. Solid-Lattice Hip Prosthesis Design: Applying Topology and Lattice Optimization to Reduce Stress Shielding from Hip Implants. Proceedings of the 2018 Design of Medical Devices Conference.

